# Intrathoracic Gastric Perforation Due to Late-Presenting Bochdalek Hernia in an Adolescent: A Rare Case of Panvisceral Herniation

**DOI:** 10.7759/cureus.106576

**Published:** 2026-04-07

**Authors:** R V Vaishnav Krishna, Rahul Khanna, Ram Niwas Meena, Malayathi Sai Venkatesh, Sri Maruthi Ravinder Raj Kaushik

**Affiliations:** 1 General Surgery, Institute of Medical Sciences, Banaras Hindu University, Varanasi, IND

**Keywords:** anterior gastric perforation, bochdalek's hernia, congenital diaphragmatic hernia (cdh), emergency nontrauma laparotomy, general gastroenterology, intrathoracic gastric perforation, multiple organ herniation, panvisceral herniation, rare case

## Abstract

Congenital diaphragmatic hernias (CDHs), particularly those of the Bochdalek type, arise when the pleuroperitoneal canal fails to close during fetal development, most often affecting the left posterolateral aspect of the diaphragm. These hernias are typically identified in the neonatal period, making their appearance in adolescence quite rare. When they do occur later in life, they may present with a range of GI or respiratory symptoms, which can complicate diagnosis. A missed or delayed diagnosis, especially in cases involving visceral perforation, can significantly increase the risk of complications and morbidity. A 15-year-old girl presented with a seven-day history of abdominal pain, multiple episodes of vomiting, shortness of breath, and obstipation. Examination revealed diminished air entry and audible bowel sounds over the left hemithorax, raising early suspicion of a diaphragmatic defect. A chest radiograph revealed complete opacification of the left hemithorax. Further evaluation with ultrasonography and contrast-enhanced CT of the thorax and abdomen demonstrated a left posterolateral diaphragmatic defect measuring approximately 9 × 6 cm. Multiple abdominal viscera, including bowel loops, omentum, stomach, spleen, and the pancreatic tail, were found to be herniating into the thoracic cavity, with a significant mediastinal shift to the right. An emergency exploratory laparotomy was performed. The herniated contents were reduced, and both the peritoneal and thoracic cavities were irrigated. A 2 × 2 cm gastric perforation was identified and repaired using Graham’s omental patch technique. A 32 Fr intercostal drainage tube was inserted into the left pleural space, and abdominal drains were placed for postoperative management. The patient had an uneventful recovery apart from postoperative pneumonia, which resolved with antibiotics. She was discharged on postoperative day 14 and remained symptom-free on follow-up. This case emphasizes the need for high clinical suspicion and timely surgical intervention in adolescent patients with atypical respiratory or GI symptoms, especially in the presence of thoracoabdominal findings.

## Introduction

Diaphragmatic hernias are most commonly diagnosed in the neonatal period and are typically congenital in origin [1]. However, in older children and adolescents, late presentations are rare and may mimic GI or respiratory disorders, often leading to delayed diagnosis [2]. Left-sided hernias are more frequent due to the protective effect of the liver on the right [3].

Kinoshita et al. [3], in their study, found the prevalence of these hernias in adulthood reaching up to 20%, but most of them are usually asymptomatic. Herniation of abdominal organs into the thoracic cavity can disrupt both respiratory mechanics and GI function. Though uncommon, complications like gastric perforation can be catastrophic if not promptly addressed. Clinical presentation may vary depending on the herniated contents and associated complications, necessitating a high index of suspicion, especially when respiratory signs coexist with abdominal symptoms [4].

Intrathoracic gastric perforation through a diaphragmatic defect is very rare but associated with a high rate of mortality when it occurs [5]. We report a case of a 15-year-old female presenting to the ED in a tertiary care center in North India with features of respiratory distress and intestinal obstruction. This case highlights a rare and life-threatening presentation of late-onset congenital diaphragmatic hernia (CDH), specifically a Bochdalek hernia, in an adolescent, with intrathoracic gastric perforation. While CDH is typically a neonatal emergency, late presentation (beyond the neonatal period) occurs in 5-30% of cases and often presents with nonspecific GI or respiratory symptoms that can lead to misdiagnosis [6].

## Case presentation

A 15-year-old female presented to the ED with complaints of abdominal pain, nonpassage of flatus and stools, vomiting, and shortness of breath for seven days. The pain was diffuse and progressive, associated with abdominal distension and multiple episodes of nonbilious vomiting. There was no history of trauma, previous surgery, or significant comorbidities.

On examination, she was tachypneic (respiratory rate: 25/min) with an oxygen saturation of 90% on a face mask. On palpation of the abdomen, generalized tenderness was present, but no guarding or rebound tenderness was noted. Chest auscultation revealed decreased air entry on the left side, and bowel sounds were audible.

**Figure 1:**
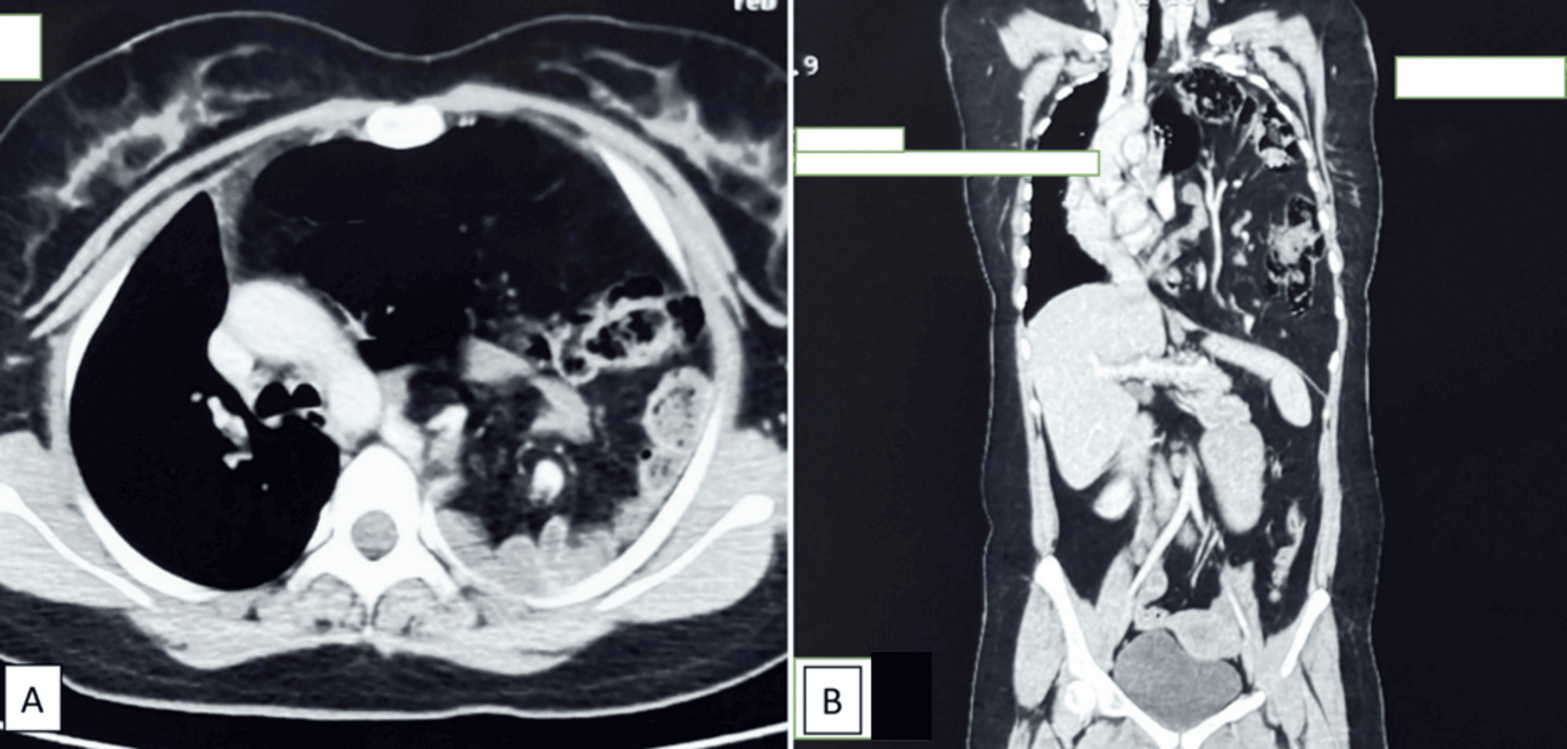
Preoperative contrast-enhanced CT scan: (A) coronal and (B) axial sections of the scan showing herniation of the abdominal contents into the left thoracic cavity

Chest X-ray showed homogeneous opacification (“white-out”) of the left hemithorax, suggestive of space-occupying pathology. Point-of-care ultrasonography suggested herniation of bowel loops and other abdominal viscera through a sizable (9 × 6 cm) left diaphragmatic defect, along with moderate fluid accumulation in both thoracic and peritoneal cavities. A CT scan (Figure 1) confirmed herniation of bowel loops, stomach, spleen, omentum, and the tail of the pancreas into the left thoracic cavity, causing a mediastinal shift to the right.

**Figure 2:**
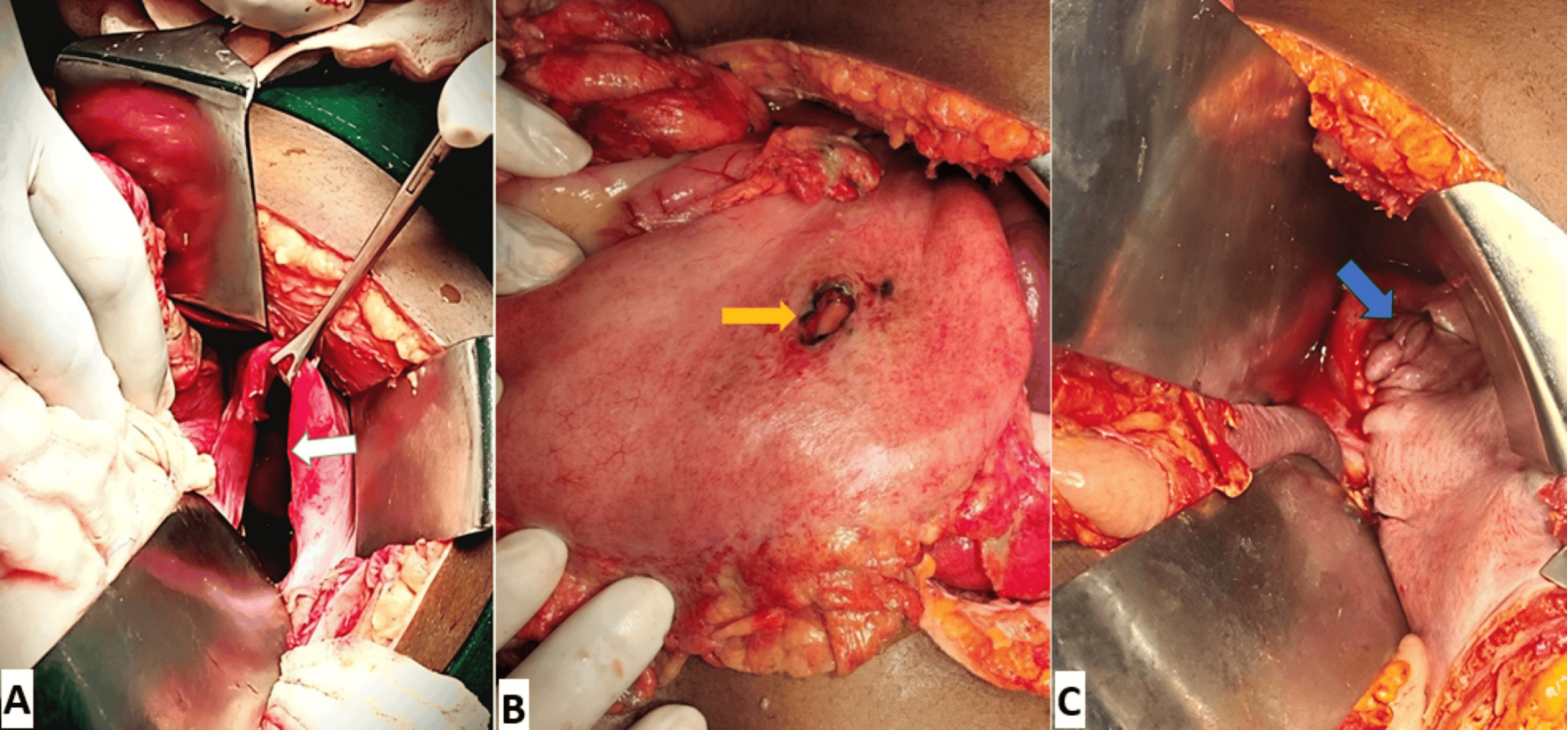
Intraoperative images showing the diaphragmatic defect (A, white arrow), the perforation in the anterior gastric wall (B, yellow arrow), and the primarily closed defect (C, blue arrow)

She was taken for an emergency exploratory laparotomy. A left subcostal incision was made. Intraoperatively, a 9 × 6 cm posterolateral diaphragmatic defect was noted, with herniation of the omentum, ileal loops, stomach, spleen, and pancreatic tail into the left thorax. The herniated viscera were gently reduced without evidence of ischemia or injury. A 2 × 2 cm perforation was noted on the anterior wall of the gastric body (Figure 2). Approximately 800 mL of serous fluid was aspirated from the left pleural cavity. Thoracic and peritoneal lavage was performed, and the perforation was repaired using Graham’s omental patch. Primary repair of the diaphragmatic defect was performed using nonabsorbable, interrupted Prolene sutures. A 32 Fr intercostal chest drain was inserted into the left hemithorax, and two abdominal drains were placed in the subhepatic and pelvic regions.

**Figure 3:**
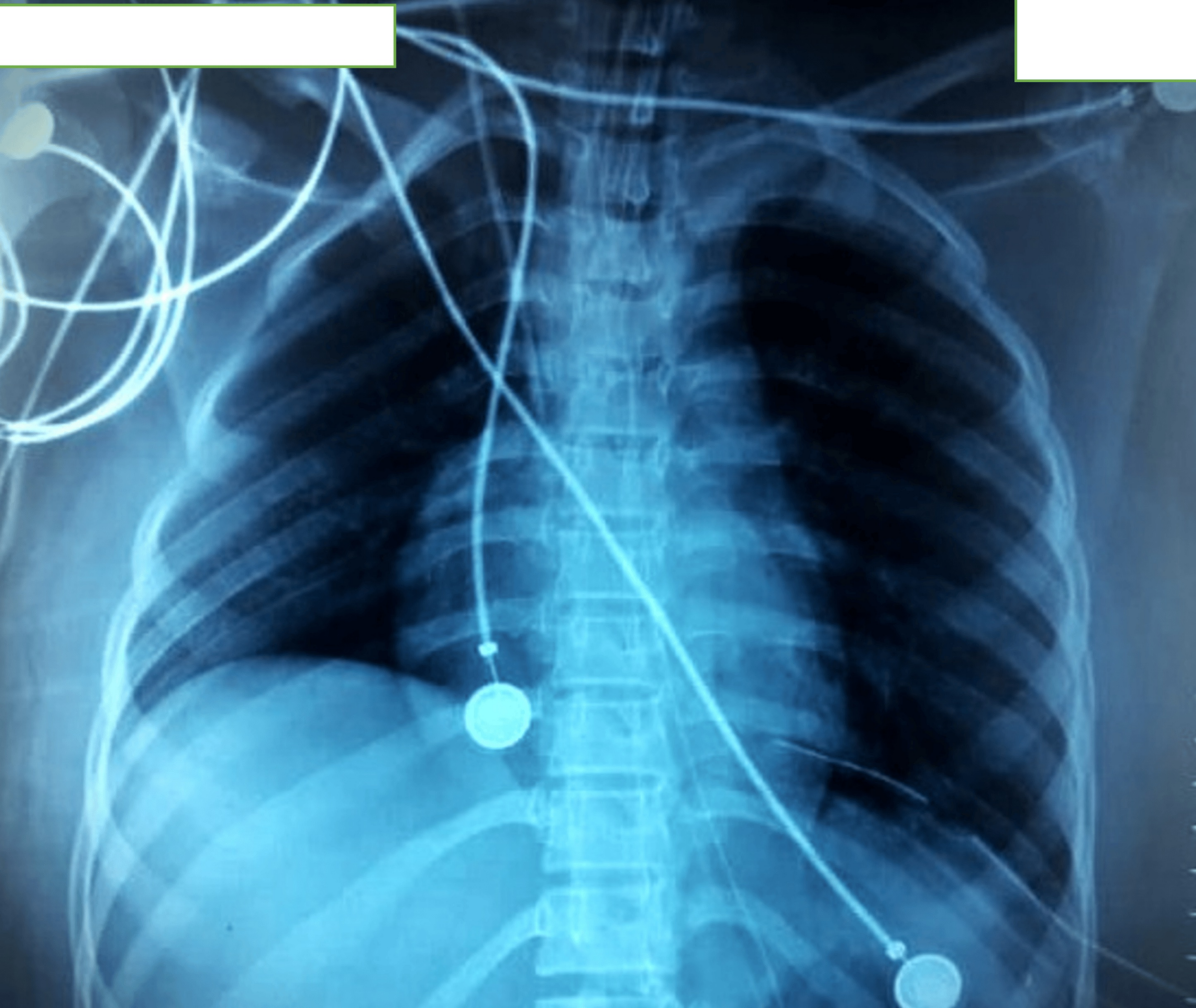
Postoperative X-ray (day 3 after operation) of the patient with a left intercostal drainage tube in situ

Postoperatively, she developed mild left basal pneumonia, managed conservatively with intravenous antibiotics and incentive spirometry. She passed flatus and stools by postoperative day 5, and her chest X-ray (Figure 3), taken on postoperative day 3, showed significant re-expansion of the lung. She was discharged on postoperative day 14 after resolution of pneumonia, normal bowel function, a healthy surgical wound, and a chest X-ray demonstrating satisfactory lung re-expansion. She was followed up with a clinical examination and chest X-ray every two weeks for three months without recurrence or respiratory complaints.

## Discussion

Diaphragmatic hernias can be classified as congenital or acquired based on the etiology. In adults, the most common cause is acquired diaphragmatic rupture secondary to trauma [7]. CDH is a developmental defect in the diaphragm that typically presents in neonates with respiratory distress. However, late presentation beyond infancy is rare, accounting for only 5-30% of CDH cases [2]. Various studies have shown that CDHs in adults are usually asymptomatic and not recognized in the preoperative period [8]. Symptoms depend on the anatomical location of the defect, with varied thoracic and abdominal manifestations. In a symptomatic case, the most common clinical features are thoracic and abdominal pain, respiratory distress, and mechanical intestinal obstruction [9]. The most common types of congenital hernias are Morgagni’s defect, which is located anteriorly, and Bochdalek’s defect, which is located posterolaterally [10]. These patients often exhibit atypical symptoms such as GI obstruction, recurrent respiratory infections, or nonspecific abdominal complaints, making diagnosis challenging. In our case, there was no history of trauma, and therefore, the posterolateral diaphragmatic defect was clearly a case of congenital Bochdalek hernia. Imaging revealed a panvisceral herniation, a finding reported in very few case studies [9,11,12]. The significant mediastinal shift further suggested a longstanding, uncorrected diaphragmatic defect.

Intrathoracic gastric perforation is an exceptionally rare and life-threatening complication of diaphragmatic hernias. Proposed mechanisms include gastric overdistension due to obstruction at the hernia neck, ischemia from volvulus or compression, or pressure necrosis [13]. In our patient, the perforation was detected intraoperatively, reinforcing the need for timely surgical exploration of suspected strangulated hernias. This rare situation has been described in case reports by Vinnicombe et al. [5] and Lim and Kostin [14]. Gedik et al. [15] observed that it can be fatal in about 32% of patients presenting with severe symptoms, which may be due to visceral strangulation, perforation, or intrathoracic complications. Although the majority are asymptomatic, there is a case in the literature that was diagnosed through hematemesis as a result of hemorrhage from the herniated fundus [7]. In another case, a diaphragmatic hernia presented with a splenic vein thrombus [16]. As evident from these reports, diaphragmatic hernias can present with unusual symptoms [17].

Surgical repair remains the definitive treatment. A left subcostal incision was preferred over midline laparotomy, as the herniated contents, including the stomach, spleen, and pancreatic tail, were predominantly located in the left upper quadrant and left hemithorax. The subcostal approach provided better exposure of the left diaphragm and facilitated safe reduction of herniated viscera, repair of the diaphragmatic defect, and management of gastric perforation. Although midline laparotomy is commonly used in emergency settings for rapid abdominal access, a subcostal incision offers more direct access to left-sided diaphragmatic pathology and is considered appropriate when pathology is localized to the left upper quadrant [18].

In our case, a primary diaphragmatic repair was feasible due to the absence of tension or contamination, and the gastric perforation was successfully repaired with Graham’s omental patch. While some authors advocate a thoracoabdominal approach in such complex cases [12,13,19], our experience suggests that exploratory laparotomy alone is sufficient when the diagnosis is clear and access to both cavities is possible through the diaphragm. In our case, the abdominal approach allowed complete visualization of herniated contents and adequate access for gastric perforation repair. The absence of dense adhesions or necrotic tissue allowed for a safe and effective laparotomy-only approach. The ability to address both intra-abdominal and thoracic pathology via the diaphragm further supports this strategy in select cases. Postoperative pneumonia is common following large hernia reductions due to lung re-expansion or preexisting atelectasis, and it should be monitored carefully. Our patient improved with conservative management and was discharged under satisfactory conditions.

This case reinforces the need to keep diaphragmatic hernia in mind when adolescents present with the unusual combination of respiratory distress and GI obstruction, especially when imaging suggests thoracoabdominal involvement. The presence of unusual herniated organs, such as the pancreas, and complications like intrathoracic gastric perforation necessitate a high index of suspicion, timely imaging, and prompt surgical intervention.

## Conclusions

Late-presenting CDH is a rare anomaly diagnosed after 30 days of age, with some cases surfacing in adolescence, often presenting with overlapping GI and respiratory symptoms. Due to these nonspecific, intermittent symptoms, it is frequently misdiagnosed initially as pneumonia or gastroenteritis. When complicated by visceral herniation and intrathoracic gastric perforation, it becomes a surgical emergency requiring prompt diagnosis and intervention. This case highlights the importance of maintaining a high index of suspicion in atypical presentations and demonstrates that timely surgical repair, even in complex herniations involving multiple viscera, can result in favorable outcomes with appropriate intraoperative and postoperative care.
